# Unraveling the Relationships between Ecosystems and Human Wellbeing in Spain

**DOI:** 10.1371/journal.pone.0073249

**Published:** 2013-09-05

**Authors:** Fernando Santos-Martín, Berta Martín-López, Marina García-Llorente, Mateo Aguado, Javier Benayas, Carlos Montes

**Affiliations:** 1 Social-Ecological Systems Laboratory, Department of Ecology, Universidad Autonoma de Madrid, Madrid, Spain; 2 Sociology and the Environment Research Area, Social Analysis Department, Universidad Carlos III, Madrid, Spain; National Institute of Water & Atmospheric Research, New Zealand

## Abstract

National ecosystem assessments provide evidence on the status and trends of biodiversity, ecosystem conditions, and the delivery of ecosystem services to society. I this study, we analyze the complex relationships established between ecosystems and human systems in Spain through the combination of Driver-Pressure-State-Impact-Response framework and structural equation models. Firstly, to operationalize the framework, we selected 53 national scale indicators that provide accurate, long-term information on each of the components. Secondly, structural equation models were performed to understand the relationships among the components of the framework**.** Trend indicators have shown an overall progressive biodiversity loss, trade-offs between provisioning and cultural services associated with urban areas *vs*. regulating and cultural services associated with rural areas, a decoupling effect between material and non-material dimensions of human wellbeing, a rapid growing trend of conservation responses in recent years and a constant growing linear trend of direct or indirect drivers of change. Results also show that all the components analyzed in the model are strongly related. On one hand, the model shows that biodiversity erosion negatively affect the supply of regulating services, while it is positively related with the increase of provisioning service delivery. On the other hand, the most important relationship found in the model is the effect of pressures on biodiversity loss, indicating that response options for conserving nature cannot counteract the effect of the drivers of change. These results suggest that there is an insufficient institutional response to address the underlying causes (indirect drivers of change) of biodiversity loos in Spain. We conclude that more structural changes are required in the Spanish institutional framework to reach 2020 biodiversity conservation international targets.

## Introduction

Ecosystem assessments offer an opportunity to learn about the contributions of nature (known as *ecosystem services*) to human wellbeing (i.e., the contributions obtained from these ecosystem services) [1]–[4]. These assessments have revealed new possibilities for analyzing the complex effects of policy decisions and human actions on the structure and processes of ecosystems, the services they provide and their consequences on human wellbeing [5], [6]. In fact, since the completion of the global Millennium Ecosystem Assessment [7], many other studies have adapted its conceptual framework to a regional, national or subnational scale by using long-term time series indicators [8], [9]. Because ecosystem assessment studies ultimately aim to analyze the effects of ecosystems and biodiversity on human wellbeing, it is necessary to understand the interrelationships of all the ecological and social components to define appropriate response options at different scales [10].

On a national scale, most of the completed assessments have focused on explaining the relationship between the state of their ecosystem services and the direct causes (i.e., pressures) of degradation. In many cases, other components, such as indirect drivers of change, have been empirically excluded from the analysis because their relations with ecosystem services are not obvious, and time series data at the scale of assessment are often absent [11]. Therefore, to analyze the intricate associations between the ecological and social components, a systematic and consistent methodology that uses indicators of social and ecological processes is needed [12].

The present study aims to evaluate direct and indirect effects that the loss of biodiversity and ecosystem services have on human wellbeing in Spain. We strive to identify the long-term (1960–2010) dynamics and interrelationships of different components of natural and social systems. We specifically (i) analyzed trends and exchange rates of individual indicators related to biodiversity, ecosystem services, human wellbeing, institutional responses, and direct and indirect drivers of change upon the component; (ii) calculated aggregated indices to better understand trends in each of those components; (iii) developed graphical conceptual depictions of trends in the indices and interrelationships of all components; and (iv) explored the causal relationships between biodiversity loss, ecosystem services, wellbeing, institutional responses, and drivers of change.

The scientific community has recently recognized the need to strengthen the incorporation of scientific knowledge into the decision-making process to properly assess the effect of nature on human wellbeing [13], [14]. For instance, scientific knowledge has also been highlighted internationally by different policy-oriented institutions [15], [16], and nationally, it has also been recently recognized by the Millennium Ecosystem Assessment of Spain [1] and the Spanish Strategic Plan on Natural Heritage and Biodiversity [17]. This study has been motivated to provide evidence on the status and trends of biodiversity, ecosystem conditions, and the delivery of ecosystem services of value to people.

## Materials and Methods

### The Driver-Pressure-State-Impact-Response (DPSIR) Framework

We used the Driver-Pressure-State-Impact-Response (DPSIR) framework to visualize the complex relationships between ecosystems and human systems in Spain from a holistic point of view. The DPSIR framework is a common approach used to explore these relationships [18], [19] because it provides an organized method for analyzing the causes, consequences and responses to changes [20], [21]. Although the DPSIR framework was first applied in the social sciences [22], it was later applied in environmental sciences [23], [24] and has recently been proposed as a method for assessing ecosystem services [9], [19], [20], [25]. However, to our knowledge, no previous national scale assessments have used the DPSIR framework to analyze the linkages between the components of ecological and human systems.

**Figure 1:**
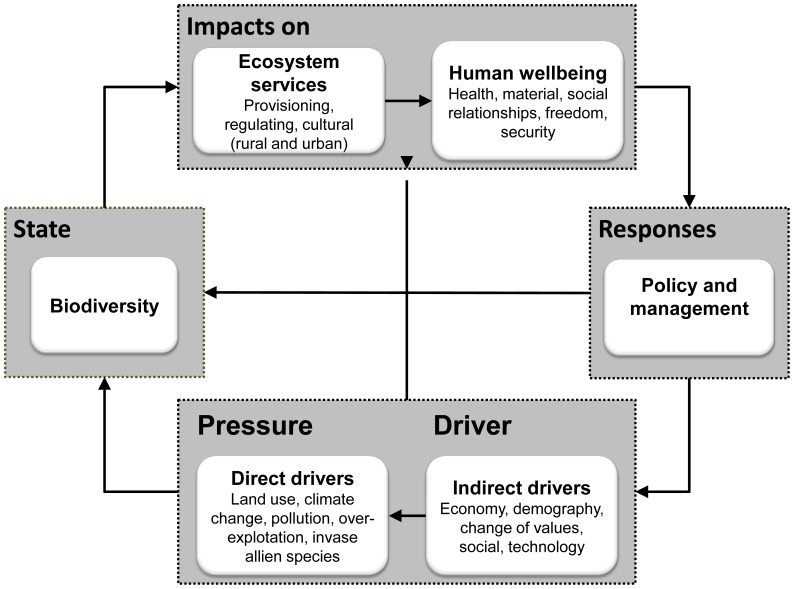
Study framework. DPSIR was used to analyze the complex relationships established between ecosystems and human systems in Spain. Modified from [20].

In this study, we adapted the DPSIR framework to analyze the connections among biodiversity loss, ecosystem services, human wellbeing and society’s responses to preserve the ecosystem service flow (Figure 1). In this context, *drivers* are the underlying factors promoting environmental change. These factors may be demographic, economic, cultural, sociopolitical or technological and are defined as indirect drivers of change in the Millennium Ecosystem Assessment framework [7]. These drivers produce different *pressures*, such as land use change, climate change, pollution, invasive alien species, and overexploitation, which may affect ecological integrity. These pressures (defined as direct drivers of change in the Millennium Ecosystem Assessment framework [7]) change the *state* of ecosystems and biodiversity thus affecting the ecosystem service delivery to society. Therefore, *impacts* can be understood as changes in both the supply of ecosystem services and human wellbeing [19], [25]. Depending on the social perception of wellbeing, governments and society perform different actions (*responses*) to control the effect of drivers or to preserve the ecosystem’s capacity to supply services.

### Indicators and Aggregate Indices

To operationalize the DPSIR framework, we selected national scale indicators that provide accurate information on each of the DPSIR components (Figure 1) and meet specific criteria. Therefore, selected indicators contained the following four characteristics: (1) indicators were understandable and widely accepted for the multiple stakeholders involved in the Spanish national ecosystem assessment; (2) indicators clearly expressed information on and sensitivities to other components of the DPSIR framework (e.g., biodiversity loss indicators should be sensitive to the intensification of changes in pressures); (3) indicators were temporally explicit such that trends could be measured over time, scalable so that they could be aggregated to different scale levels, and quantifiable so that the information obtained could be easily compared [9]; and (4) indicators contained official statistical datasets from 1960 through 2010 [8].

Overall, 53 indicators were selected and were related to biodiversity loss (1 indicator), ecosystem services (20 indicators) (i.e., provisioning (9 indicators), regulating (7), and cultural services (5)), human wellbeing (10 indicators), policy responses (4 indicators), indirect drivers (10 indicators) and pressures (8 indicators). Supporting information (Tables S1–S6) summarizes the following DPSIR components for each of the selected indicators: the source of the indicator data, the unit of measurement, the period of time used for the analysis (based on the available data), and a graphical representation of the evolution of indicator trends.

To explore the trend evolution of each components of the DPSIR framework, we standardized each indicator by subtracting the mean of the indicator value and dividing by the standard deviation. Once the indicators were standardized, we calculated the trends for the time series of each indicator based on the slope from a linear regression. Moreover, we categorized the trends of each indicator as follows: (1) *highly improve* (↑↑) was when the slope of the regression models was higher than 0.08; (2) *improve* (↑) was when the slope of the regression models was between 0.08 and 0.04; (3) *stable* (↔) was when the slope of the regression models was between 0.04 and −0.04; (4) *decline* (↓) was when the slope of the regressions was negative and between the values of −0.04 and −0.08; and (5) *highly decline* (↓↓) was when the slope of the regressions was lower than −0.08.

We then aggregated the indicators in indices for each of the components of the DPSIR framework: (1) biodiversity loss, (2) ecosystem services based on the classification presented in [26] (i.e., provisioning services, regulating services, cultural urban services, and cultural rural services), (3) human wellbeing including material and non-material dimension, (4) policy responses, (5) direct drivers of change or pressures, and (6) indirect drivers of change. Except for biodiversity loss (i.e., the Red List Index), the aggregation rule used was the geometric mean, which is a useful method for benchmarking because it reduces the compensability of poor performance in specific indicators by including the high values in others [27].

### Unraveling Relationships between Ecosystems and Human Wellbeing

**Figure 2:**
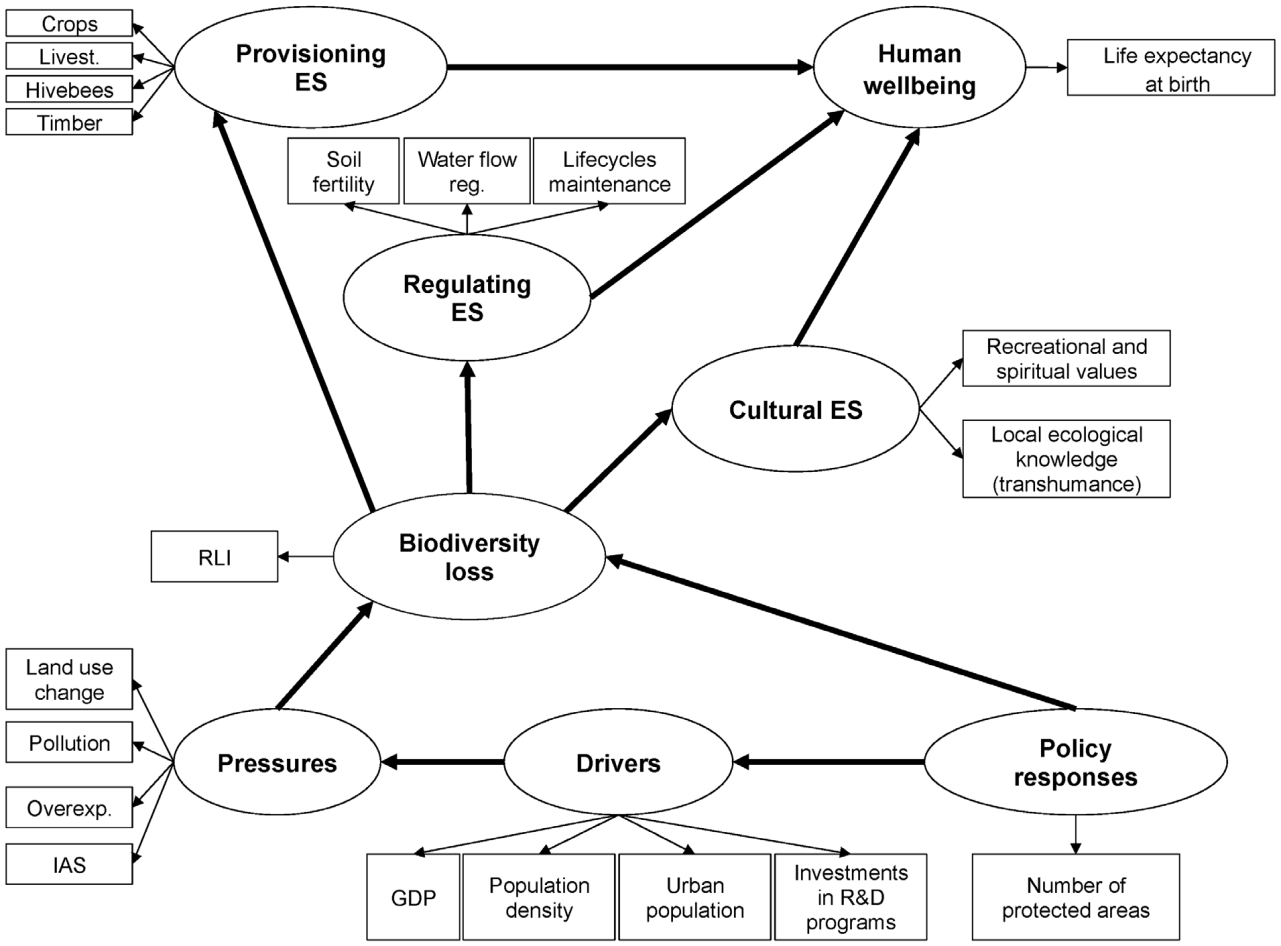
Diagram of a conceptual structured equation model describing the potential relationships between the components of the DPSIR framework. Latent variables (which are the components of the DPSIR framework) are showed in ellipses, while manifest variables (which are the indicators used) are presented in rectangles. (RLI = Red List Index; IAS = invasive alien species; GDP = Gross Domestic Product; R&D = research and development; ES = ecosystem services).

We explored the relationships between biodiversity loss, ecosystem services, human wellbeing, drivers of change (both direct and indirect) and policy responses using structural equation modeling (SEM) [28], [29]. SEM is used for describing conceptual models and estimating the links among their variables, where some latent variables, linked by linear regressions, are measured by sets of manifest variables [30]. We developed a model that presents the hypothesized relationships among latent variables (i.e., biodiversity loss, provisioning, regulating and cultural services, human wellbeing, policy responses, drivers and pressures) in a path diagram (Figure 2). In this model, policy responses was considered as exogenous variable, which did not depend on the other variables; while biodiversity loss, ecosystem services, human wellbeing and drivers of change were considered as endogenous variables, modeled as being dependant of other variables. Then, as manifest variables, we selected the most representative indicators of which we have dataset from all the analyzed period (i.e., 1960–2010) (Figure 2). Each of SEM involves two types of relationships: (1) relationships among the latent variables –i.e., the structural model- and (2) the links between each latent variable and its own block of manifest variables –i.e., measurement model- [29]. The Dillon-Goldstein’s rho index was used to test the block unidimensionality of manifest variables in each of the latent variables [31].

We assessed the prediction capability of the model using different indices: (1) the adjusted *R* ^2^ values of each relation in the structural model, (2) the average communality index which measures the capability of each latent variable in explaining the variances of the manifest variables and thus evaluate the measurement model [32] and (3) the goodness of fit index which measures the quality of the whole model [33].

XLSTAT software by Addinsoft was used to perform statistical analysis.

## Results

### Trends of Individual Indicators

#### Biodiversity and ecosystem services

Overall, there has been a continued decline in the biodiversity of vertebrates in Spain over the past three decades (Table 1). Of the 20 selected indicators for analyzing ecosystem services, 9 showed declining trends. Regulating services are the most affected group with all indicators portraying a losing trend. In particular, water quality seems to be the most affected regulating service (Table 1). In contrast, some provisioning (i.e., terrestrial and freshwater plants and animals for food and biotic materials) and cultural services associated with urban demand (i.e., recreational activities and environmental education) have improved in Spain over the last decades (Table S2).

**Table 1:** Trend of biodiversity status (particularly vertebrates) based on the Red List Index [55] and ecosystem services using national scale indicators (classification based on the CICES system [56]).

Biodiversity			Indicator			Trend	
Vertebrates	Mammals, Birds, Reptiles, Amphibians and Fish		The Red List Index			↓↓	
Ecosystem service	Group	Class			Indicator		Trend
**Provisioning**							
Nutrition	Terrestrial plants and animals for food	Crops		Total production of cereals, fruits and olive			**↑**
		Livestock		Total production of meat			**↑**
		Wild plants and animals and their products		Number of hive bees of *Apis melifera*			**↑**
	Freshwater plants and animals for food	Aquaculture product		Total production of aquiculture			**↑**
Water supply	Water for human consumption	Domestic water use		Water for human use			**↓**
Materials	Biotic materials	Non-food vegetal fibers		Total timber production			**↑**
				Total paper pulp production			**↑**
				Total agricultural fibers production			**↔**
Energy	Renewable abiotic	Hydropower production		Total production of hydropower			**↔**
**Regulating**							
Regulation of physic-chemical environment	Water quality regulation	Water purification and oxygenation		Volume of wastewater treated			**↓↓**
	Pedogenesis and soil quality	Maintenance of soil fertility		Fertilizer consumption in arable lands			**↓**
Flow regulation	Air flow regulation	Microclimatic regulation		Total CO_2_ emissions			**↓**
	Water flow regulation	Attenuation of runoff and discharge rates		Damages paid by floods			**↓**
Regulation of biotic environment	Pest and disease control	Biological control mechanisms		Number of invasive alien plants			**↓**
Regulation against hazards	Lifecycles maintenance	Habitat refuges		Total number of forest fires			**↓**
**Cultural**							
Symbolic	Recreation and spiritual community activities	Sacred places or species		Number of pilgrims to Santiago			**↑**
Intellectual and experiential		Landscape character for recreational opportunities		Number of visitors to protected areas			**↑↑**
	Information & knowledge	Environmental educational		Number of equipment for environmental education			**↑↑**
		Local ecological knowledge		Number of sheep in transhumance system			**↓**
				Traditional cork production			**↓**

#### Human wellbeing

Indicators selected to independently analyze the five dimensions of human wellbeing (i.e., health, material, security, freedom and social relations) revealed different temporal trends (Table 2). Health dimension indicators showed that while physical health (i.e., life expectancy and infant survival rate) has continued to improve year after year in Spain, mental health has declined. The material dimension, which is the standard of living or welfare measured by economic indicators, increased in conjunction with the evolution of gross domestic product (GDP) per capita and the total material requirements of the Spanish population. Although the indicator used to assess security (deaths by natural accidents) has shown an overall increase since 1960, the trend is non-linear, which implies that this indicator varies in relation to natural hazards (e.g., droughts, floods, etc.) (Table S3). The freedom of action and choice portrays a significant improvement due to important increases in education and civil liberties. In contrast, the social relations dimension showed a clear decline based on the social cohesion and time availability indicators.

**Table 2:** Trend of human wellbeing indicators divided into its five dimensions.

Dimension	Sub-dimension	Indicator	Trend
Health	Physical	Life expectancy at birth	**↑**
		Infant survival rate	**↑**
	Mental	Revers of suicides/100,000 inhabitants	**↓**
Material	Livelihoods	Gross domestic product per capita	**↑**
	Access to goods	Total material requirement	**↑**
Security	Natural hazards	Number of deaths by natural accidents	**↓**
Freedom of choice and actions	Education	Percentage of literacy	**↑↑**
	Civil liberties	Civil liberty index	**↑**
Social relationships	Time availability	Revers of television consumption	**↓↓**
		Number of holidays and non-working days	**↓↓**

#### Response options

During the last two decades, Spain has made significant environmental management efforts, particularly in regards to the following: (i) conservation policies to control biodiversity loss (i.e., declaration of protected areas and creation of conservation programs for endangered species); (ii) social participation in environmental issues (i.e., volunteers to national parks) and (iii) environmental market initiatives such as the organic agriculture production. Most of these response options have grown rapidly in the last three decades (Table 3 and Table S4) although the positive impacts are not yet reflected on biodiversity loss or ecosystem services indicators (Table 1).

**Table 3:** Trend of response options indicators to determine the efforts performed by institutions in Spain in relation to environmental issues.

Class	Type	Indicator	Trend
Conservation	Biodiversity conservation	Number of species conservation programs	**↑↑**
		Number of protected areas	**↑**
	Environmental engagement	Number of volunteers to national parks	**↔**
Social participation on environmental issues			
Environmental markets initiatives	Organic agricultural production	Organic agriculture surface	**↑↑**

#### Drivers

Economic drivers indicate important economic development (GDP per capita) in the last decades, although with key variations in stabilization and equity distribution (total employment) (Table 4 and Table S5). Demographic and cultural indicators suggest a conversion from rural to urban society, with associated ways of life, during the last decades. In particular, there has been a constant increase in population density and urban growth, indicating that rural abandonment has constantly increased in the past five decades. Associated transformation of demographic and cultural aspects are, for example, reducing the number of children per woman and increasing the maternity age to nearly 31 years old (Table S5). Sociopolitical indicators show that although Spain has gained social liberties in the last decades, there is a growing sense of unconformity with the political system. For example, the number of demonstrations has increased from approximately 5,000 people in 1982 to more than 25,000 in 2009 (see Table S5). According to scientific production, there has been an important recession on scientific investments in Spain in the last years, but over the last five decades, there has been an increase in the number of publications on environmental issues.

**Table 4:** Trend of drivers and pressures indicators to determine the main causes and effects on biodiversity and ecosystems in Spain.

Class	Type	Indicator	Trend	
**Drivers**				
Economic	Economic development	Total gross domestic product		**↑**
		Total employment		**↓**
Demographic	Population density	Average human density		**↑**
	Fertility rate	Number of children per woman		**↓**
	Maternity age	Age of the mother at delivery first baby		**↑**
Cultural	Urban population	Population living in municipalities with more than 10.000 inhabitants		**↑**
Sociopolitical	Credibility on political system	Vote abstention		**↔**
	Conformity on political system	Number of total demonstrations		**↑**
Scientific	Investments	Percentage of the ratio investment/GDP		**↑↑**
	Scientific production	Total number of publications		**↑↑**
**Pressures**				
Land-use change	Urbanization	Number of initiate houses		**↑↑**
	Habitat fragmentation	New kilometers of railroads		**↑↑**
Climate change	Emissions	Greenhouse gases		**↑↑**
	Glaciers	Surface cover by permanent glaciers		**↓↓**
Over-exploitation	Biotic materials	Marine species extracted		**↑**
	Abiotic materials	Groundwater extracted for irrigation		**↑**
Invasive alien species	Invasive plants	Number of invasive plants		**↑**
Pollution	Air pollution	Total CO_2_ emissions		**↑**

#### Pressures

All indicators of pressure (direct drivers of change) have continuously increased in the past decades (Table 4 and Table S6). During recent decades, there has been a linear trend, indicating a constant and increasing pressure over the natural capital of Spain (i.e., surfaces of permanent glaciers have been reduce in 80% during the last three decades, emissions of carbon dioxide have experienced an increase of 510% since 1960, and extraction of groundwater for irrigation has increased by more than 600 times). However, land use change in terms of the urbanization (i.e., number of initiated houses) has been the most evident and perturbing pressure, especially in coastal and inland water ecosystems, over Spanish natural systems during the last five decades.

### Trend of Aggregated Indices

**Figure 3:**
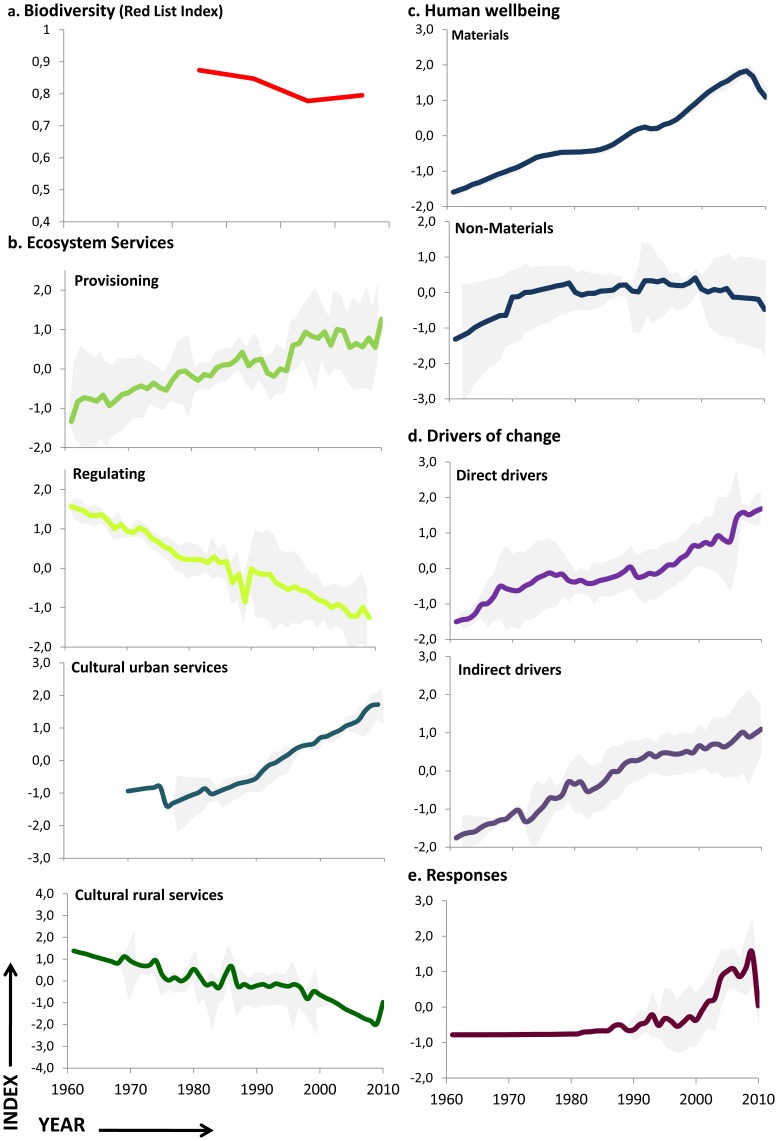
Aggregate indices for each of the DPSIR components evaluated. (a) **Biodiversity** based on Red List Index of vertebrates, (b) **Ecosystem services** based on ten indicators of provisioning services, seven indicators of regulating services and seven in cultural services (four associated more with urban demand and three with rural people), (c) **Human wellbeing** using twelve indicators (two for material dimension and ten for other non-material dimensions) (d) **Responses options** using five indicators related to conservation strategies, social participation and market initiatives (e) **Drivers** (indirect drivers of change) based on ten indicators related to the demographic, economic, cultural, sociopolitical and scientific aspects (f) **Pressures** (direct drivers of change) based on eight indicators related to land use, climate change, over-exploitation, pollution and invasive alien species. The data are standardized according to the arithmetic mean corresponding to the time series of 1960 through 2010. The shading indicates the variability of the data with a 95% confidence interval for each of the aggregated indices. For details information regarding individual indicators, see Table S1–S6.

Indicators for each of the components assessed were aggregated in indices to better understand general trends and possible trade-offs among them (Figure 3). The results show that conservation strategies primarily based on the creation of protected areas and species conservation programs are not sufficient to address the loss of biodiversity (Figure 3a) and degradation of regulating and cultural services related to rural contexts (Figure 3b). In fact, this issue seems to indicate a relationship between increased trends from pressures and drivers (Figure 3e and 3f) and the loss of biodiversity and degradation of these types of ecosystem services (Figure 3b). However, provisioning services and cultural services mainly associated with urban demand have increased since 1980 (Figure 3b). With regard to human wellbeing (Figure 3c), there is a clear decoupling effect between standard of living (the material dimension) and quality of life (aggregation of health, social relations, security and freedom dimensions).

Trends of aggregated indices also show variations in some components since the beginning of the current crisis in 2007. For example, while the material dimension of human wellbeing (Figure 3c) and the response options (Figure 3d) have suffered a significant decline, cultural services demanded by urban people have become more linear (Figure 3b), and those demanded by rural people have started to recover. The trends of drivers and pressures indices (Figure 3e and 2f) appear not to have changed, but their slopes have softened; however, despite the crisis, these indicators follow slow patterns (slow variables).

Additionally, the variability in the data (the shadow area behind indices of Figure 3) represents the range for each of the aggregated indices. This level of uncertainty in the data can be used to predict future trends. Interestingly, the variability in the regulating and provisioning services (Figure 3b) is expanding in similar proportion to the pressures and drivers (Figure 3e and 3f).

### Relationships among the Components of the DPSIR Framework

**Figure 4:**
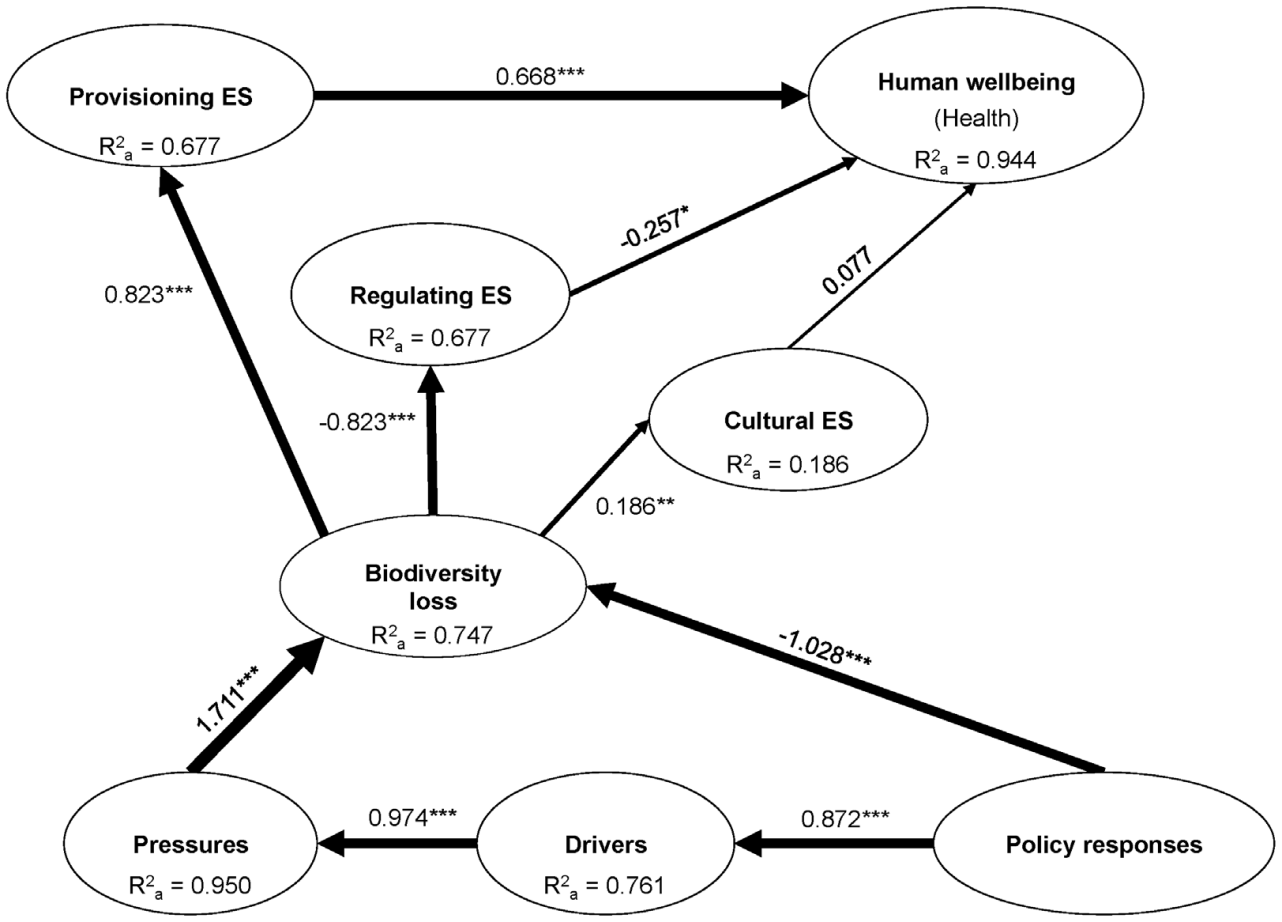
Structured equation model results indicating the relationships between latent variables. Width of arrows is proportional to the strength of path coefficients and numbers near arrow lines denote the standardized regression coefficients. Significance levels are as follows: *p≤0.05, **p≤0.01 and ***p≤0.001. (ES = ecosystem services).

The SEM results are shown is Figure 4, meanwhile the indices used for assess the predictable capacity of the model are shown in Table 5. The SEM model obtained is able to explain greater than two-third of the variance (adj *R^2^*≥0.677) in all of the latent variables, except for cultural services (adj *R^2^* = 0.186). The low variance explained in the case of cultural services can be due to the manifest variables correspond to those cultural services demanded by urban people (i.e., recreational and spiritual values) and by rural people (i.e., local ecological knowledge), which follow opposite trends (Figure 3b).

**Table 5:** Results from the SEM performed.

Latent variable	D.G. Rho*	Adjusted *R* ^2^	Average communality
1. Biodiversity loss	1.000	0.747	1.000
2. Provisioning services	0.968	0.677	0.883
3. Regulating services	0.880	0.677	0.710
4. Cultural services	–	0.186	0.947
5. Human wellbeing	1.000	0.944	1.000
6. Drivers (indirect drivers)	0.985	0.761	0.942
7. Pressures (direct drivers)	0.975	0.950	0.831
8. Policy responses	1.000	–	1.000
Goodnes of fit index = 0.915			

* D.G. rho: Dillon Goldstein’s rho index. A block of manifest variables is considered homogeneous if the index is higher than 0.7.

All the components analyzed in the model are strongly related (Figure 4). Pressures have the largest effect on biodiversity loss (*β* = 1.711), followed by policy options (*β* = −1.028). However, they have the opposite effect as pressures have a positive effect on biodiversity loss (i.e., increasing rates of pressures causes increasing rates of biodiversity loss), while policy options have a negative effect (i.e., increasing rates of creation of protected areas reduces biodiversity loss rates). The model also shows a strong positive causal relation between drivers and pressures (*β* = 0.974). In addition, whereas biodiversity loss has a positive effect on the delivery of provisioning services (*β* = 0.823), it produces a deterioration of regulating services (*β* = −0.823). There is also a weak positive significant link between biodiversity loss and the delivery of cultural services (*β* = 0.186). However, we should keep in mind that the correlation between both manifest variables included in cultural services is negative (*r = *−0.908; *p*<0.0001), indicating the inconsistency of this latent variable, as well as the potential negative effect of biodiversity loss on those cultural services demanded by rural people. Finally, we do not found a clear link between ecosystem services and the health dimension of human wellbeing because it seems that only provisioning services are positively related with the life expectancy at birth indicator (*β* = 0.668).

## Discussion

### Efforts to Manage Biodiversity Loss are Faced with the Challenge of Managing Both Drivers and Pressures

**Figure 5:**
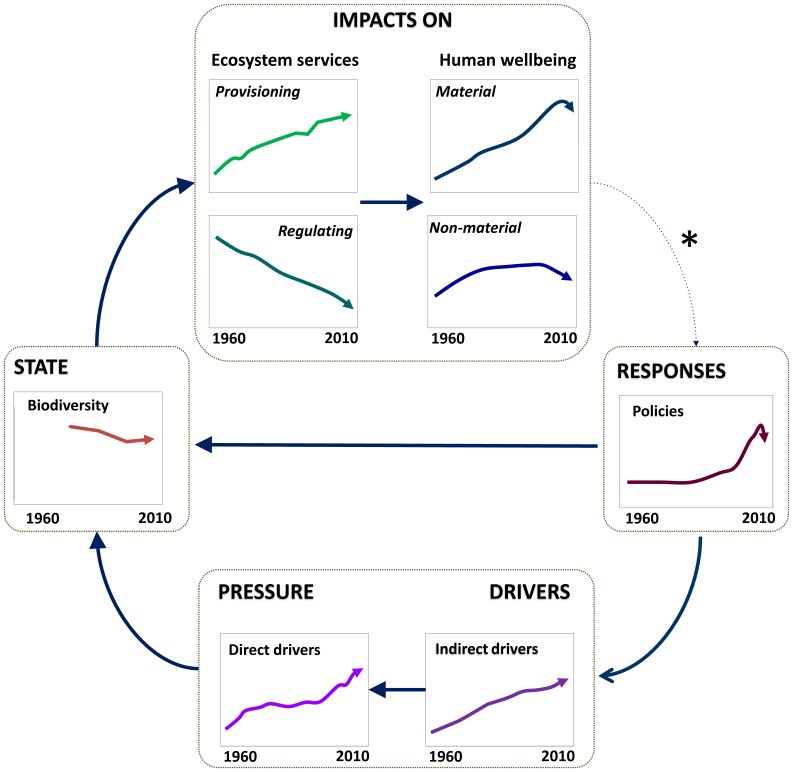
Integrative results from conceptual framework. It represents trend evolution of aggregate indices and relationship among the DPSIR components based on SEM results *The arrow between human wellbeing and responses is with dotted line because it was not analyzed in the study. (Inspired in [35]).

Loss of biodiversity and the deterioration of certain ecosystem service flows is a result of numerous drivers acting synergistically [15], [34]. Results from this study showed that Spanish biodiversity loss has been increasing even though the conservation efforts are increasing during the last decades. This pattern has also been recognized worldwide based on the various publications that demonstrated our failure to meet the 2010 biodiversity target [12], [15], [35], [36]. SEM results showed that these political efforts have focused mainly on responding to direct effect (pressures), although there was a weaker response to address indirect drivers probably because their associations with ecosystems are more complex and require higher institutional changes. Results also show a strong positive causal relationship between drivers and pressures suggesting that efforts to control biodiversity loss are faced with the challenge of managing both drivers and pressures. We summarize these results in a conceptual diagram where trend and main relationships of each DPSIR component can be shown (Figure 5).

The main policies for conserving biodiversity in Spain have been mostly focused on designing protected areas that promote the conservation of the habitat of threatened species, as well as on the legislation regarding endangered species (i.e., the National Catalogue of Threatened Species). In fact, since 1960, the national network has consistently expanded such that the current network includes 1712 designated protected areas covering 61892 km^2^; this expansion is similar to the global trends of protected areas [35], [37]. In the case of either Spanish protected areas or the National Catalogue of Threatened Species, the primary objective has usually been to promote the conservation of the habitat of a particular species, mostly species of mammals and birds [38], [39]. However, recent literature has concluded that the components of biodiversity that ensure the delivery of ecosystem services are functional diversity and species diversity of taxonomic groups of microorganisms, vegetation or invertebrates [5], [40]–[42]. Therefore, the main strategies of biodiversity conservation do not include conservation of these key components that ensure the capacity to deliver ecosystem services. To resolve this issue, land-use planning decisions should be used to support previous efforts and guide indirect drivers of change such as demographic, cultural and sociopolitical aspects.

Paradoxically, in the context of the current economic crisis, indices trends (Figure 3) show that since 2007, we have been witnessing variations in the drivers of change, which clearly show that the current economic crisis has some positive effects on the Spanish nature. This result reinforces the idea that the underlying causes of biodiversity loss are the drivers because although response options have significantly declined in recent years, biodiversity and some ecosystem services are recovering within this new socio-economic situation. This situation opens a window of opportunities where new conservation strategies should be managed in coordination with other sectorial policies at different organizational scales [35] and should consider the key components of biodiversity that guarantee the ecosystem service delivery.

### Decoupling Effect between Material and Non-material Dimensions of Human Wellbeing

Our results also show how the loss of biodiversity and degradation of some ecosystem services has a direct impact on human wellbeing (Figure 5). Although the selection of indicators associate to human wellbeing were difficult to find and therefore the relationships among them are difficult to explain, indicators trends highlight a lifestyle that promotes the material dimension (economic growth or GDP) and extensive consumption of materials but fails to increase non-material dimension of human wellbeing in basic as social relations or security [37], [38]. This lifestyle is prevalent in many occidental countries, and similar results have been reported in different studies [44]–[47]. For example, although the average wages doubled in the U.S. between 1957 and 2002 and increased 5.4 times in Japan between 1958 and 1988, the reported happiness of inhabitants remained fairly constant in both countries [48]. In concurrence with our results, recent studies related ecosystem services to domains of human wellbeing that could serve as the foundation for developing an index of wellbeing for the U.S. [49].

Results from SEM highlighted that human wellbeing (live expectancy) has been mainly accomplished through provisioning services (Figure 4). For example, economic growth associated with the highest per capita consumption has led to increased demand for services such as more than 7 times the consumption of meat, approximately 4 times the use of fertilizer, and more than 16 times the production of paper [43] (Table S1–S2). This lifestyle, mainly in urban areas, has increased the potential of pressures and drivers with negative consequences on biodiversity and on the services directly dependent on biodiversity [5], [40], thus contributing to the transformation of rural practices and lifestyles.

Results are consistent with the rural-urban gradient regarding the social perceptions for cultural services identified by Martín-López et al. [26], which are most likely the consequence of different lifestyles and socio-economic characteristics. Moreover, the intensification of provisioning services and cultural services associated with urban demand promotes social changes affecting the system of beliefs, local identity, and worldview of rural populations [50], which involves the erosion of regulation and cultural services associated with rural areas [26]. In fact, Spanish rural areas are undergoing two parallel processes: depopulation and intensification of agro-pastoral practices, resulting in both the loss of local knowledge and traditional management activities that are essential for ecosystems and biodiversity conservation [26], [51]. Managing the balance between the rural and urban systems will largely determine the socio-ecological future of Spain. Promoting a migratory flow between rural and urban areas that favors job opportunities, education and quality of life and a reconnection of urban society with ecological systems [52] could be one way to improve non-material dimensions of human wellbeing in Spain.

### Moving Forward to 2020 Biodiversity Targets in Spain

The significance of biodiversity for human wellbeing was recognized 20 years ago with the formation of the Convention on Biological Diversity. The failure to meet the 2010 biodiversity target [15] stimulated a set of new targets for 2020 (the Aichi targets) and, in conjunction, governments have been negotiating the establishment of a new assessment body, the Intergovernmental Science-Policy Platform on Biodiversity and Ecosystem Services (IPBES). In Europe, the EU Biodiversity strategy calls on member States to map and assess the state of ecosystems and their services in the national territory [16]. At the national scale, the Strategic Plan on Natural Heritage and Biodiversity [17] recognizes the social role of ecosystems and biodiversity due to their influence on human health and quality of life but also because of their contribution to social and economic development through the supply of essential ecosystem services. It emphasizes the social and economic value of ecosystem services and the importance of their inclusion in policies. In fact, our results show the close association between biodiversity, ecosystem services and human wellbeing, illustrating that biodiversity erosion can negatively affect the delivery of regulating and cultural services. These results are in line with other global evaluations [12], [40], [41].

In order to counteract the biodiversity and ecosystem services degradation in Spain, significant efforts for both the science and policy circles need to be made in the next few years if the Aichi targets are to be met [40]. As highlighted by Perrings *et al.* (2010), the first strategic goal to meet 2020 targets is to “*address underlying causes of biodiversity loss by mainstreaming biodiversity across government and society”* [14]. Our results have identified that there is insufficient institutional response to address these underlying causes (indirect drivers of change) in Spain, and we believe that responses are necessary to fill this political gap and to reach the 2020 targets. To meet these targets, more structural changes are required that recognize biodiversity as a global public service that integrate biodiversity conservation into economic and landscape policies [47], [53]. Additionally, unsustainability patterns require cultural changes in society as a whole and individually [54].

## Concluding Remarks

This study includes the first national scale analysis to assess the effect of biodiversity loss on human wellbeing of the Spanish population. We present a useful methodology to assess complex relationships between nature and social systems using a complex analysis of indicators’ trends through SEM. In fact, results have increased our understanding of: (i) temporal trends of biodiversity loss, ecosystem service delivery and human wellbeing, as well as their relation with the effect of multiple drivers of change, pressures and response options at national scale; (ii) how all the DPSIR components analyzed are strongly related (Figure 4), illustrating the close relationships between biodiversity, ecosystem services, human wellbeing, and drivers of change; (iii) there exists a strong positive causal relation between drivers and pressures, indicating the robust relationships between indirect and direct drivers of change; (iv) the most important relationship found in the SEM model is the effect of pressures on biodiversity loss (Figure 4), indicating that response options for conserving nature cannot counteract the effect of the direct drivers of change; (v) current conservation strategies are not sufficient to address biodiversity loss and degradation of regulating and cultural services related to rural contexts (Figure 3); (vi) a clear relationship was found between biodiversity loss and ecosystem services delivery in which while biodiversity loss has a positive effect on the delivery of provisioning services, it causes a deterioration of regulating services (Figure 4); (vii) it was identified a clear trade-off between provisioning and cultural services associated with urban areas *vs*. regulating and cultural services associated with rural areas; and finally, (viii) in the context of the current economic crisis, indices trends show that since 2007, we have been witnessing variations in the drivers of change, which clearly show that the current economic crisis has some positive effects on the Spanish biodiversity (Figure 5). Therefore, in the current context of social, economic and ecological crisis, we have a window of opportunity open to rethink conservation programs from a more holistic point of view that ultimately will have better impacts on natural systems and our human wellbeing.

## Supporting Information

Table S1 **Biodiversity indicator description and evolution based on the Red list Index of threatened species of vertebrates in Spain.**
(DOCX)

Table S2 **Ecosystem services indicators description and evolution for its three types: provisioning, regulating and cultural.**
(DOCX)

Table S3 **Human wellbeing indicators description and evolution for its five dimensions: material, health, security, freedom and social relations.**
(DOCX)

Table S4 **Response options indicators description and evolution from Spanish institution dealing with environmental issues.**
(DOCX)

Table S5 **Drivers indicators description and evolution that indirectly affect biodiversity and ecosystems in Spain.**
(DOCX)

Table S6 **Pressures indicators description and evolution that directly affect biodiversity and ecosystems in Spain.**
(DOCX)
