# Supplementary material for: Unraveling the Relationships between Ecosystems and Human Wellbeing in Spain
Source: PLoS One. 2013 Sep 5;8(9):e73249. doi: 10.1371/journal.pone.0073249 (PMC3764230; doi:10.1371/journal.pone.0073249)
Supplement: Table S1 — Biodiversity indicator description and evolution based on the Red list Index of threatened species of vertebrates in Spain. (DOCX) [file pone.0073249.s001.docx]

**Table S1. Biodiversity indicator description and evolution based on the Red list Index** [1] **of threatened species of vertebrates in Spain.**

| **Taxonomic group** | **Indicator description** | **Indicator evolution** |
| --- | --- | --- |
| Vertebrates | The Red List Index of fish, amphibians and reptiles, birds and mammals | 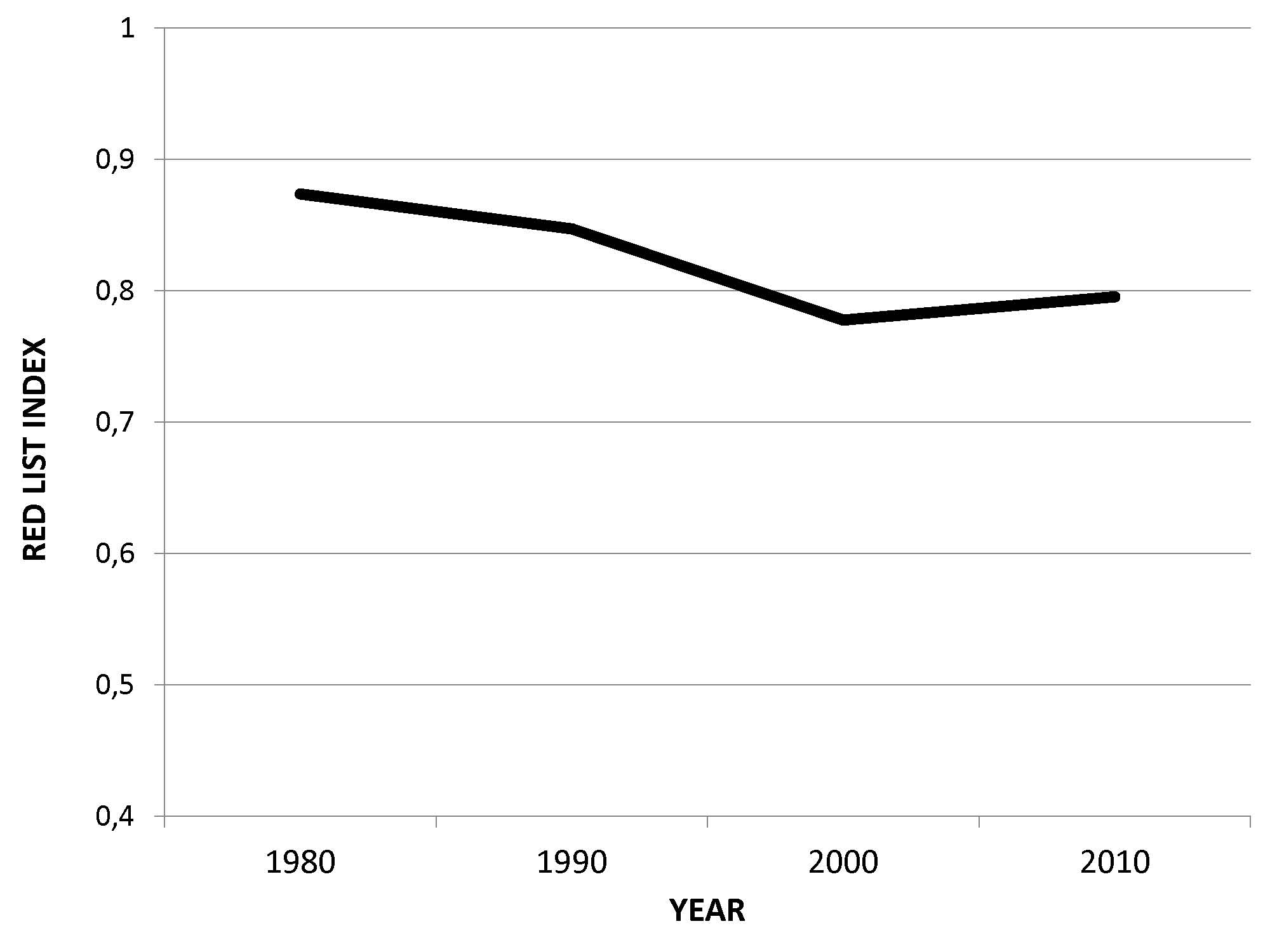 |
|  | Period: 1980-2010 |  |
|  | Units: Dimensionless |  |
|  | Source: [2,3,4,5,6,7,8] |  |

**REFERENCES**

1. Blanco JC, González JL (1992) Libro Rojo de los Vertebrados de España. ICONA. Madrid
2. Bubb PJ, Butchart SHM, Collen B, Dublin H, Kapos V, Pollock C, Stuart SN, Vié JC (2009) IUCN Red List Index: Guidance for National and Regional Use. Gland, Switzerland.
3. Doadrio I (2001) Atlas y Libro Rojo de los Peces Continentales de España. National Museum of Natural Science. General Direction of Nature Conservation. Madrid.
4. ICONA (1986) Lista roja de los vertebrados de España. ICONA, Ministerio de Agricultura, Pesca y Alimentación, Madrid.
5. Martí R, Moral JC (2003) Atlas de las Aves Reproductoras de España. Madrid, Spain: The Spanish Ministry of the Environment (Autonomous Organism of Nature Reserves) and Spanish Ortnithologist Society.
6. Morales J, Lizana M (2011) El estado de la biodiversidad de los vertebrados españoles. Causa de la riqueza de especies y actualizacion taxonómica. Memorias R.Soc.Esp.Hist.Nat., 9: 285-342
7. Palomo JL, (2007) Atlas y Libro Rojo de los mamíferos terrestres de España. Madrid, Spain: The Spanish Ministry of the Environment. Autonomous Organism of Nature Reserves.
8. Pleguezuelos JM, Márquez R (2004) Atlas y Libro Rojo de los Anfibios y Reptiles de España. Madrid, Spain: National Museum of Natural Science. General Direction of Nature Conservation.
