# Supplementary material for: Unraveling the Relationships between Ecosystems and Human Wellbeing in Spain
Source: PLoS One. 2013 Sep 5;8(9):e73249. doi: 10.1371/journal.pone.0073249 (PMC3764230; doi:10.1371/journal.pone.0073249)
Supplement: Table S2 — Ecosystem services indicators description and evolution for its three types: provisioning, regulating and cultural. (DOCX) [file pone.0073249.s002.docx]

**Table S2. Ecosystem services indicators description and evolution for its three types: provisioning, regulating and cultural.**

| **Ecosystem Services** | **Indicator description** | **Indicator evolution** |
| --- | --- | --- |
| **Provisioning** | | |
| **Nutrition** | | |
| Crops | Total production of cereals, fruits and olive |  |
|  | Period: 1961-2010 |  |
|  | Units: Tons |  |
|  | Source: [1] |  |
| Livestock | Total production of meet |  |
|  | Period: 1961-2010 |  |
|  | Units: Tons |  |
|  | Source: [1] |  |
| Aquiculture | Total production of aquiculture |  |
|  | Period: 1961-2010 |  |
|  | Units: Tons |  |
|  | Source: [1] |  |
| Wild plants and animals and their products | Number of hivebees of *Apis melifera* |  |
|  | Period: 1961-2008 |  |
|  | Units: Nº hivebees |  |
|  | Source: [1] |  |
| **Water supply** | | |
| Water for human consumption | Water harvesting for human use | 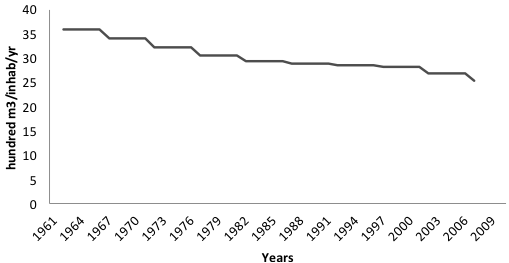 |
|  | Period: 1962-2007 |  |
|  | Units: m^3^ per inhabitant and year |  |
|  |  |  |
|  | Source: [1] |  |
| **Biotic materials** | | |
| Timber | Total wood production |  |
|  | Period: 1961-2009 |  |
|  | Units: Millions m^3^ |  |
|  | Source: [1] |  |
| Paper | Total paper pulp production |  |
|  | Period: 1961-2010 |  |
|  | Units: Millions tons |  |
|  | Source: [1] |  |
| Fibers | Total agricultural fibers production |  |
|  | Period: 1961-2010 |  |
|  | Units: Thousand tons |  |
|  | Source: [1] |  |
| **Energy** | | |
| Hydropower | Total production of hydro power |  |
|  | Period: 1961-2008 |  |
|  | Units: Millions tons of oil equivalent (toe) |  |
|  | Source: [2] |  |
| **Regulating** | | |
| **Regulation of physico-chemical environment** | | |
| Water quality regulation | Volume of wastewater treated |  |
|  | Period: 1990-2010 |  |
|  | Units: m^3^ per inhabitant and year |  |
|  | Source: [4] |  |
| Maintenance of soil fertility | Fertilizer consumption |  |
|  | Period: 1961-2007 |  |
|  | Units: kilograms per hectare of arable land |  |
|  | Source: [3] |  |
| **Flow regulation** | | |
| Air flow regulation | Total CO_2_ emissions |  |
|  | Period: 1961-2008 |  |
|  | Units: Millions metric tons of CO_2_ |  |
|  | Source: [3] |  |
| Water flow regulation | Damages paid by floods by insurance companies |  |
|  | Period: 1971-2007 |  |
|  | Units: Thousand of expedients per year |  |
|  | Source: [5] |  |
| **Regulation of biotic environment** | | |
| Biological control mechanisms | Number of invasive alien plants |  |
|  | Period: 1961-2003 |  |
|  | Units: number of invasive alien plants |  |
|  | Source: [6] |  |
| **Regulation against hazards** | | |
| Lifecycles maintenance | Number of forest fires |  |
|  | Period: 1961-2008 |  |
|  | Units: Thousands of forest fires per year |  |
|  | Source: [4] |  |
| **Cultural** | | |
| **Symbolic** | | |
| Recreational and spiritual values | Number of pilgrims to Santiago |  |
|  | Period: 1970-2008 |  |
|  | Units: Thousands of pilgrims |  |
|  | Source: [8] |  |
| **Experiential** | | |
| Recreation and community activities | Number of visitors to protected areas |  |
|  | Period:1976-2009 |  |
|  | Units: Visitors to protected areas |  |
|  | Source: [9] |  |
| **Information & knowledge** | | |
| Environmental education | Number of equipment for environmental education |  |
|  | Period: 1983-2005 |  |
|  | Units: Number of equipments |  |
|  | Source: [4] |  |
| Local ecological knowledge | Traditional cork production | 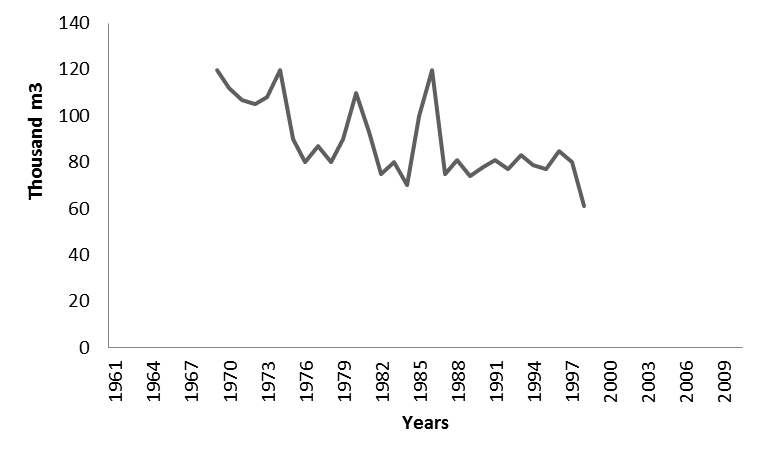 |
|  | Period: 1969-1998 |  |
|  | Units: Thousands of m^3^ |  |
|  | Source: [4] |  |
| Local ecological knowledge | Number of sheeps in transhumance |  |
|  | Period: 1961-2009 |  |
|  | Units: number of sheeps |  |
|  |  |  |
|  | Source: [4] |  |

**REFERENCES**

1. Faostat (2011) Food and agriculture organization of the United Nations. Statistical division. Available online (visited November 2011): (<http://faostat.fao.org/>)

2. International Energy Agency (2011) World energy statistic. Available online (visited November 2011) (<http://www.iea.org/stats/index.asp>)

3. World Bank (2011) World Bank data by country. Available online (visited November 2011) <http://data.worldbank.org/country/spain>

4. Spanish Ministry of Agriculture Food and Environment (2011) Anuraio de estadistica. Perfil ambiental de España. Available online (visited November 2011) <http://www.magrama.gob.es/es/estadistica/temas/default.aspx>

5. Insurance Compensation Consortium (2011) Available online (visited November 2011) <http://www.consorseguros.es/web/guest/i>

6. Sanz Elorza M, Sanchez ED, Sobrino Vesperina E, (2004) Atlas de las plantas alóctonas invasoras en España. Dirección General para la Biodiversidad. Madrid, 384 p.

7. Spanish National Statistical Institute. Available online (visited November 2011) <http://www.ine.es/>

8. Office of Sociology the Archdiocese of Santiago de Compostela (2011) Available online (visited November 2011) <http://peregrinossantiago.es/esp/>

9. Europac. 2010. Anuario EUROPARC-España del estado de los espacios naturales protegidos 2010. FUNGOBE. Madrid. 104

10. Isi Web of Knowledge (2011) Available online (visited November 2011) [http://apps.webofknowledge.com/UA_](http://apps.webofknowledge.com/UA_GeneralSearch_input.do?product=UA&search_mode=GeneralSearch&SID=S1H4m3J@NDBIPB7H32K&preferencesSaved)
