# Supplementary material for: Unraveling the Relationships between Ecosystems and Human Wellbeing in Spain
Source: PLoS One. 2013 Sep 5;8(9):e73249. doi: 10.1371/journal.pone.0073249 (PMC3764230; doi:10.1371/journal.pone.0073249)
Supplement: Table S3 — Human wellbeing indicators description and evolution for its five dimensions: material, health, security, freedom and social relations. (DOCX) [file pone.0073249.s003.docx]

**Table S3. Human wellbeing indicators description and evolution for its five dimensions: material, health, security, freedom and social relations.**

| **Human wellbeing** | **Indicator description** | **Indicator evolution** | |
| --- | --- | --- | --- |
| **Material / Livelihoods** | | | |
| GPD per capita | Gross domestic product per capita |  | |
|  | Period: 1961-2008 |  |  |
|  | Units: Thousands of $ PPP |  |  |
|  | Source: [1] |  |  |
| **Material / Access to goods** | | | |
| Total material requirement | Physical materials that are mobilized each year to support an economy |  | |
|  | Period: 1961-2010 |  |  |
|  | Units: Ton per inhabitant |  |  |
|  | Source: [2] |  |  |
| **Health / Physical** | | | |
| Life expectancy at birth | The average numbers of years a newborn child would life if current mortality patterns were to stay the same | 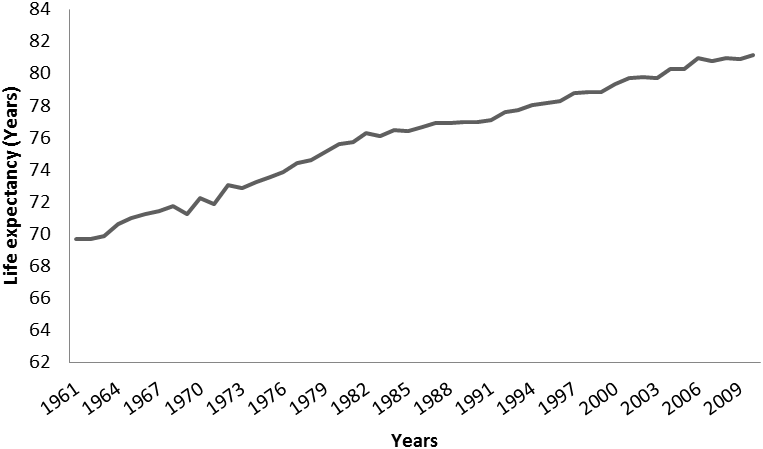 | |
|  | Period: 1961-2010 |  |  |
|  | Units: years |  |  |
|  | Source: [1] |  |  |
| Infant mortality rate | Number of deaths in children under 5 year/1000 child born | **** | |
|  | Period: 1961-2008 |  |  |
|  | Units: Number of deaths in children under 5 year/1000 child born |  |  |
|  | Source: [1] |  |  |
| **Health / Mental** | | | |
| Suicides | Number of suicides/100000 inhabitants | 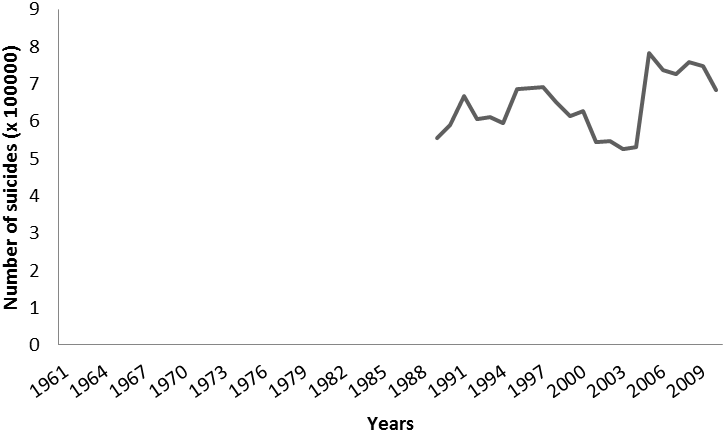 | |
|  | Period: 1989-2010 |  |  |
|  | Units: Thousands of suicides |  |  |
|  | Source: [3] |  |  |
| **Security** | | | |
| Deaths by natural disasters | Number of deaths by natural disasters | 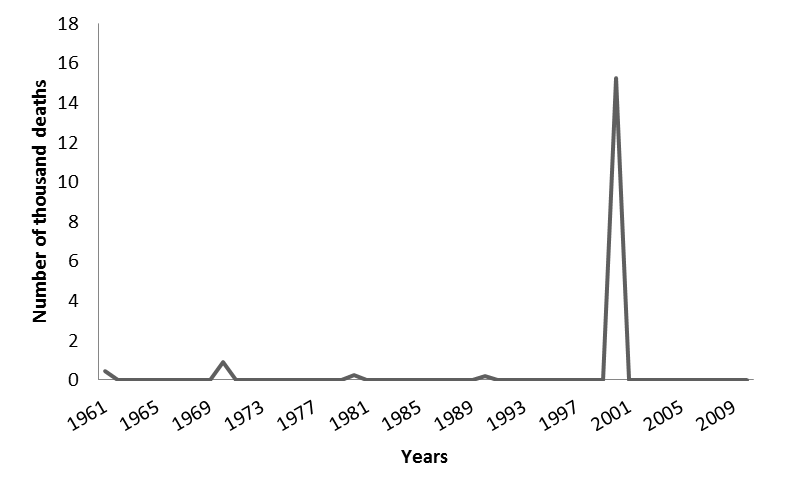 | |
|  | Period: 1961-2000 |  |  |
|  | Units: Thousands of deaths |  |  |
|  | Source: [4] |  |  |
| **Freedom of choice and actions** | | | |
| Education | Percentage of illiteracy from total population | |  |
|  | Period: 1989-2009 | |  |
|  | Units: % of illiteracy | |  |
|  | Source: [3] | |  |
| Civil liberties index | Quantify the freedoms of expression and belief, associational and organizational rights, rule of law, and personal autonomy without interference from the state. | | **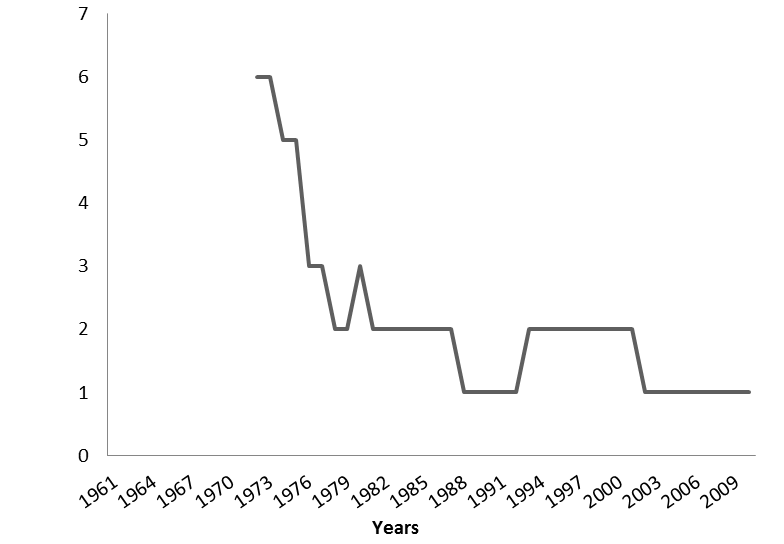** |
|  | Period: 1972-2010 | |  |
|  | Units: From 1 to 7 (1 indicates the highest degree of freedom and 7 the lowest level) | |  |
|  | Source: [6] | |  |
| **Social relationships / Cohesion** | | | |
| Television consumption | Average time spent watching television |  | |
|  | Period: 1991-2010 |  |  |
|  | Units: min/day |  |  |
|  | Source: [7] |  |  |
| **Social relationships / Time availability** | | | |
| Holidays and non-working days | Percentage of annual hours non worked regarding an effective working day |  | |
|  | Period: 1991-2008 |  |  |
|  | Units: % |  |  |
|  | Source: [8] |  |  |

**REFERENCES**

1. World Bank (2011) World Bank data by country. Available online (visited November 2011) <http://data.worldbank.org/country/spain>

2. Carpintero O (2005). El metabolismo de la economía española. Recursos naturales y huella ecológica (1955-2000). Fundación César Manrique.

3. Spanish National Statistical Institute. Available online (visited November 2011) <http://www.ine.es/>

4. International Disaster Database (2011) Available online (visited November 2011) <http://www.emdat.be/>

5. The Institute of Women, Spanish Ministry of Health, Social Services and Equality (2011) Available online (visited November 2011) <http://www.inmujer.es/estadisticas/portada/home.htm>

6. Freedom House (2011) Available online (visited November 2011) <http://www.freedomhouse.org/>

7. Barlovento Comunication (2011) Available online (visited November 2011) <http://www.barloventocomunicacion.es/>

8. Spanish Ministry of Employment and Social Security (2011) Available online (visited November 2011) <http://www.empleo.gob.es/index.htm>
