# Supplementary material for: Unraveling the Relationships between Ecosystems and Human Wellbeing in Spain
Source: PLoS One. 2013 Sep 5;8(9):e73249. doi: 10.1371/journal.pone.0073249 (PMC3764230; doi:10.1371/journal.pone.0073249)
Supplement: Table S4 — Response options indicators description and evolution from Spanish institution dealing with environmental issues. (DOCX) [file pone.0073249.s004.docx]

**Table S4. Response options indicators description and evolution from Spanish institution dealing with environmental issues.**

| **Response options** | **Indicator description** | **Indicator evolution** |
| --- | --- | --- |
| **Biodiversity conservation** | | |
| Number of species conservation programs | Number of programs approved regarding species including in the Threatened Species Catalog | **** |
|  | Period: 1989-2010 |  |
|  | Units: number of programs |  |
|  | Source: [1] |  |
| Number of protected areas | Total number of protected areas declared | **** |
|  | Period: 1962-2010 |  |
|  | Units: number of protected areas |  |
|  | Source: [1] |  |
| **Social participation** | | |
| Volunteers | Number of volunteers to national parks | **** |
|  | Period: 2000-2010 |  |
|  | Units: number of volunteers |  |
|  | Source: [2] |  |
| **Market initiatives** | | |
| Organic agriculture | Organic agriculture area | **** |
|  | Period: 1994-2010 |  |
|  | Units: Thousands of hectares |  |
|  | Source: [1] |  |

**REFERENCES**

1. Spanish Ministry of Agriculture Food and Environment (2011) Anuraio de estadistica. Perfil ambiental de España. Available online (visited November 2011) <http://www.magrama.gob.es/es/estadistica/temas/default.aspx>

2. Europac. 2010. Anuario EUROPARC-España del estado de los espacios naturales protegidos 2010. FUNGOBE. Madrid. 104
