# Supplementary material for: Unraveling the Relationships between Ecosystems and Human Wellbeing in Spain
Source: PLoS One. 2013 Sep 5;8(9):e73249. doi: 10.1371/journal.pone.0073249 (PMC3764230; doi:10.1371/journal.pone.0073249)
Supplement: Table S5 — Drivers indicators description and evolution that indirectly affect biodiversity and ecosystems in Spain. (DOCX) [file pone.0073249.s005.docx]

**Table S5. Drivers indicators description and evolution that indirectly affect biodiversity and ecosystems in Spain.**

| **Drivers** | **Indicator description** | **Indicator evolution** |
| --- | --- | --- |
| **Economic** | | |
| Total GDP | Gross domestic product | **** |
|  | Period: 1961-2008 |  |
|  | Units: Millions of $ PPP |  |
|  | Source: [1] |  |
| Total unemployment | Unemployment of total labor force | **** |
|  | Period: 1980-2010 |  |
|  | Units: % |  |
|  | Source: [1] |  |
| **Demographic** | | |
| Population density | Human population density | **** |
|  | Period: 1961-2010 |  |
|  | Units: Persons per squared kilometer |  |
|  | Source: [1] |  |
| Fertility rate | Number of children that would be born to a woman over her lifetime | **** |
|  | Period: 1961-2010 |  |
|  | Units: Children per woman |  |
|  | Source: [1] |  |
| Maternity age | Age of the mother at time of delivery first baby | **** |
|  | Period: 1975-2010 |  |
|  | Units: Years |  |
|  | Source: [2] |  |
| **Sociopolitical** | | |
| Vote abstention | Participants not going to vote | **** |
|  | Period: 1977-2010 |  |
|  | Units: % of people |  |
|  | Source: [2] |  |
| Number of total demonstrations | Total number of demonstrations | **** |
|  | Period: 1982-2007 |  |
|  | Units: Number of demonstrations |  |
|  | Source: [3] |  |
| **Cultural** | | |
| Urban population | Population living in municipalities with more than 2000 inhabitants | **** |
|  | Period: 1961-2010 |  |
|  | Units: % |  |
|  | Source: [2] |  |
| **Scientific and technological** | | |
| Investments in R&D programs | Investment in R&D programs | **** |
|  | Period: 1967-2009 |  |
|  | Units: % (from GDP) |  |
|  | Source: [2] |  |
| Scientific production | Total number of publications | **** |
|  | Period: 1981-2006 |  |
|  | Units: number of publications |  |
|  | Source: [4] |  |

**REFERENCES**

1. World Bank (2011) World Bank data by country. Available online (visited November 2011) <http://data.worldbank.org/country/spain>

2. Spanish National Statistical Institute. Available online (visited November 2011) <http://www.ine.es/>

3. Spanish Ministry of Employment and Social Security (2011) Available online (visited November 2011) <http://www.empleo.gob.es/index.htm>

4. Isi Web of Knowledge (2011) Available online (visited November 2011) [http://apps.webofknowledge.com/UA](http://apps.webofknowledge.com/UA_GeneralSearch_input.do?product=UA&search_mode=GeneralSearch&SID=S1H4m3J@NDBIPB7H32K&preferencesSaved)
