# Supplementary material for: Unraveling the Relationships between Ecosystems and Human Wellbeing in Spain
Source: PLoS One. 2013 Sep 5;8(9):e73249. doi: 10.1371/journal.pone.0073249 (PMC3764230; doi:10.1371/journal.pone.0073249)
Supplement: Table S6 — Pressures indicators description and evolution that directly affect biodiversity and ecosystems in Spain. (DOCX) [file pone.0073249.s006.docx]

**Table S6. Pressures indicators description and evolution that directly affect biodiversity and ecosystems in Spain.**

| **Pressures** | **Indicator description** | **Indicator evolution** |
| --- | --- | --- |
| **Land use change** | | |
| Urbanization | Number of initiate houses | **** |
|  | Period: 1970-2009 |  |
|  | Units: Thousands of initiated houses |  |
|  | Source: [1] |  |
| Habitat fragmentation | New railroads | **** |
|  | Period: 1980-2010 |  |
|  | Units: Thousand of Kilometers |  |
|  | Source: [2] |  |
| **Over exploitation / Biotic materials** | | |
| Marine species extracted | Captures of salmons in Spanish rivers | **** |
|  | Period: 1961-2005 |  |
|  | Units: Millions of tons |  |
|  | Source: [3] |  |
| **Over exploitation / Abiotic materials** | | |
| Groundwater consumption | Groundwater extracted for irrigation | **** |
|  | Period: 1961-2004 |  |
|  | Units: Hectometers |  |
|  | Source: [4] |  |
| **Climate change** | | |
| Greenhouse gases emissions | Total greenhouse gases emisssions | **** |
|  | Period: 1990-2008 |  |
|  | Units: Tons of CO_2_ equivalent |  |
|  | Source: [4] |  |
| Glaciers | Surface cover by permanent glaciers | **** |
|  | Period: 1982-2009 |  |
|  | Units: Hectares |  |
|  | Source: [5] |  |
| **Invasive alien species** | | |
| Invasive alien species | Number of invasive plants | **** |
|  | Period: 1961-2003 |  |
|  | Units: number of species |  |
|  | Source: [6] |  |
| **Pollution** | | |
| Pollution | Total CO2 emissions | **** |
|  | Period: 1961-2010 |  |
|  | Units: Millions of tons |  |
|  | Source: [7] |  |

**REFERENCES**

1. Spanish National Statistical Institute. Available online (visited November 2011) <http://www.ine.es/>

2. World Bank (2011) World Bank data by country. Available online (visited November 2011) <http://data.worldbank.org/country/spain>

3. Sport fishing groups (2011) Available online (visited November 2011) <http://www.vadeando.com/inicio/;>

4. Spanish Ministry of Agriculture Food and Environment (2011) Anuraio de estadistica. Perfil ambiental de España. Available online (visited November 2011) <http://www.magrama.gob.es/es/estadistica/temas/default.aspx>

5. European Environment Agency (2011) ERHIN Program. Available online (visited November 2011) <http://www.eea.europa.eu/soer/countries/es/national-and-regional-story-spain>

6. Sanz Elorza M, Sanchez ED, Sobrino Vesperina E, (2004) Atlas de las plantas alóctonas invasoras en España. Dirección General para la Biodiversidad. Madrid, 384 p.

7. Carbon Dioxide Information Analysis Center (2011) Available online (visited November 2011) <http://cdiac.ornl.gov/>
